# Analysis and Implementation of an Electronic Laboratory Notebook in a Biomedical Research Institute

**DOI:** 10.1371/journal.pone.0160428

**Published:** 2016-08-01

**Authors:** Santiago Guerrero, Gwendal Dujardin, Alejandro Cabrera-Andrade, César Paz-y-Miño, Alberto Indacochea, Marta Inglés-Ferrándiz, Hima Priyanka Nadimpalli, Nicola Collu, Yann Dublanche, Ismael De Mingo, David Camargo

**Affiliations:** 1 Gene Regulation, Stem Cells and Cancer Programme, Centre for Genomic Regulation (CRG), The Barcelona Institute for Science and Technology, Barcelona, Spain; 2 Information and Communications Technology Department, Centre for Genomic Regulation (CRG), The Barcelona Institute for Science and Technology, Barcelona, Spain; 3 Instituto de Investigaciones Biomédicas, Facultad de Ciencias de la Salud, Universidad de las Américas, Quito, Ecuador; 4 Universitat Pompeu Fabra (UPF), Barcelona, Spain; 5 Universitat de Barcelona (UB), Barcelona, Spain; UGent / VIB, BELGIUM

## Abstract

Electronic laboratory notebooks (ELNs) will probably replace paper laboratory notebooks (PLNs) in academic research due to their advantages in data recording, sharing and security. Despite several reports describing technical characteristics of ELNs and their advantages over PLNs, no study has directly tested ELN performance among researchers. In addition, the usage of tablet-based devices or wearable technology as ELN complements has never been explored in the field. To implement an ELN in our biomedical research institute, here we first present a technical comparison of six ELNs using 42 parameters. Based on this, we chose two ELNs, which were tested by 28 scientists for a 3-month period and by 80 students via hands-on practical exercises. Second, we provide two survey-based studies aimed to compare these two ELNs (PerkinElmer Elements and Microsoft OneNote) and to analyze the use of tablet-based devices. We finally explore the advantages of using wearable technology as ELNs tools. Among the ELNs tested, we found that OneNote presents almost all parameters evaluated (39/42) and both surveyed groups preferred OneNote as an ELN solution. In addition, 80% of the surveyed scientists reported that tablet-based devices improved the use of ELNs in different respects. We also describe the advantages of using OneNote application for Apple Watch as an ELN wearable complement. This work defines essential features of ELNs that could be used to improve ELN implementation and software development.

## Introduction

The increasing adoption of digital technology in scientific research has led to an inevitable transition from traditional paper laboratory notebooks (PLNs) to digital ones. Electronic laboratory notebooks (ELNs) are replacing PLNs across the pharmaceutical industry and, to a lesser extent, academic research. In the past few years, the number of ELNs has increased considerably from open-source software solutions to commercial ones.

Rubacha *et al*. [1] reviewed 35 ELNs commercially available and grouped them into five categories according to their primary market audience (research and development, quality assurance/quality control, Biology, Chemistry, and multidiscipline). 20 ELNs were classified in research and development category (e.g., LABtrack) but only two of them present multidisciplinary features (PerkinElmer E-Notebook and IDBS E-WorkBook). This classification also reveals a balance in the ELN design complexity from multidisciplinary ELNs to domain-specific solutions (e.g., Studylog for animal research studies).

Recently, to expand the choice of multidisciplinary ELNs, Day *et al*. [2] improved a generic ELN [3] by introducing plug-in based chemistry functionalities. Also, Voegele *et al*. [4] developed an open-source multidisciplinary ELN based on WordPress system. In addition, non-scientific digital notebooks such as Evernote and Microsoft OneNote have been successfully implemented as ELNs [5,6]. Despite detailed analysis of ELNs [1,5–8], a survey-based study comparing their characteristics has not been documented so far.

The recent increase in use of touchscreen technology has further stimulated the development of ELN software to improve its functionalities in tablet-based devices [9,10]. This will contribute to the transition from PLNs to ELNs by giving the users a physical support to record and visualize data. Wearable technology could also enhance the intended use of ELNs by providing immediate data access during experimentation (e.g., protocols). However, no study has directly explored the usage of tablet-based devices nor wearable technology as ELN complements.

With an aim to implement an ELN in our biomedical research institute, we performed a technical comparison of six ELNs and conducted two survey-based studies to compare two ELNs. In addition, use of tablet-based devices and wearable technology as ELN complements was tested.

## Materials and Methods

### ELN evaluation

Evernote Free and Premium versions (Version 5.8.6.7519), Microsoft OneNote 2013 (Version 15.0.4763.1000 for Windows) linked to on-premise Microsoft SharePoint 2013, PerkinElmer E-Notebook and Elements and Dassault Systèmes BIOVIA Notebook were technically evaluated based on 5 main parameters including 42 subcategories. Free trial software licenses were obtained for all ELNs.

### Survey-based studies

Following technical evaluation, we selected two ELNs (PerkinElmer Elements and Microsoft OneNote linked to on-premise Microsoft SharePoint 2013) to be tested in two survey-based studies.

First, 28 scientists from 8 different laboratories volunteered for a 3-month pilot study: 17 tested only Microsoft OneNote, 5 only Elements and 6 both programs. Before the pilot study, training courses were lectured by Microsoft OneNote and Elements experts. During the testing period all experiments were recorded electronically by the volunteers. In addition, the participants used 20 iPads and 4 tablet-laptop hybrids during the study.

To evaluate both ELNs and to study the usage of tablet-based devices, group and individual questions were included. Group questions were answered by each laboratory after analysis and discussion of both solutions. Flexibility, Collaboration, Ease of searching, Image editing and Accessibility parameters were rated by the volunteers using a 5-point scale (Excellent-Good-Average-Fair-Poor).

Second, a two days full-time workshop on ELNs was conducted for an independent group of 80 students using OneNote and Elements. Students were asked to evaluate the aforementioned parameters of both programs via hands-on practical exercises. For example, students evaluated Ease of searching by finding text in pre-made experiments/notes or rated Image editing by working with agarose gels images. The above-mentioned 5-point scale was also used to rate both programs.

We also explored the functionalities of Apple Watch as a wearable ELN tool using Microsoft OneNote application.

## Results and Discussion

### ELN selection

We chose 5 ELNs based on the literature and previous experience from other biomedical research institutes. Thus, we select two non-scientific electronic notebooks, Evernote Free and Premium versions and Microsoft OneNote linked to on-premise Microsoft SharePoint 2013 due to their successful implementation in academic research (Machina & Wild, 2013; Barber *et al*., 2009). Moreover, the Max Delbrück Center for Molecular Medicine (Germany) has effectively implemented Microsoft OneNote as an ELN solution within a highly collaborative environment. We also selected two scientific ELNs, PerkinElmer E-Notebook and Elements. According to Rubacha *et*. *al*. (2011), E-Notebook is suitable for general science functionalities with specific applications in Biology compared with 33 electronic notebooks. Moreover, the E-Notebook has been successfully implemented at Flanders Institute for Biotechnology (Belgium). Due to the success of this ELN, we also chose Elements, a new web-based solution also developed by PerkinElmer. To broaden the analysis, we also include Dassault Systèmes BIOVIA Notebook.

### ELN evaluation

ELNs were technically evaluated based on recommendations and conclusions of Machina & Wild (2013) and Walsh & Cho (2013). Thus, 5 main parameters including 42 subcategories were studied (Table 1). Overall, OneNote presents almost all subcategories evaluated (39/42), followed by Evernote Premium version (31/42), BIOVIA Notebook (30/42), E-Notebook (28/42), Evernote Free version (26/42) and Elements (23/42). The evaluation of these parameters are detailed below.

**Table 1:** ELNs evaluation.

Evaluation Parameters			Evernote Free	Evernote Premium	Onenote	Elements	E-Notebook	BIOVIA Notebook
**Ease of searching**	Text in pages/notes		yes	yes	yes	yes	yes	yes
	Text in images (Optical Character Recognition, OCR)		yes	yes	yes	no	no	yes
	Text in pdf files		yes (1)	yes	yes (2)	yes	yes	yes
	Text in scientific files		no	no	no	no	no	yes (7)
	Text in microsoft office files	Word	no	yes	yes (2)	no	yes	yes
		PowerPoint	no	yes	yes (2)	no	yes	yes
		Excel	no	yes	yes (2)	no	yes	yes
	Text in illustrator files		no	no	yes (2)	yes	yes	no
**Flexibility**	Ability to link other pages/notes		yes	yes	yes	yes	yes	yes
	Ability to drag and drop		yes	yes	yes	no	yes	yes
	Ability to create your own workflow design		yes	yes	yes	no	yes	yes
	Drawing and recording	Freehand drawing	no	no	yes	no	yes	no
		Audio recording	yes	yes	yes	yes	yes	no
		Video recording	no	no	yes	yes	yes	no
	Image editing		yes	yes	yes	yes	yes	yes
	Microsoft Office files embedding	Word	no	no	yes	no (4)	yes	yes
		PowerPoint	no	no	yes	Yes	yes	yes (8)
		Excel	no	no	yes	yes	yes	yes
**Collaboration**	Ability to share pages/notes		yes	yes	yes	yes	yes	yes
	Activity constantly updated in shared notes		yes	yes	yes	yes	yes	yes
	Presentation mode for meetings		no	yes	yes	no	no	no
	Sharing modes	Can view	yes	yes	yes	yes	yes	yes
		Can edit	yes	yes	yes	yes	yes	yes
		Can edit and invite	yes	yes	no	yes	yes	yes
	Accessibility	Windows version	yes	yes	yes	yes (3)	yes	yes (9)
		Mac version	yes	yes	yes	yes (3)	no	yes (3)
		Linux	yes (3)	yes (3)	yes (3)	yes (3)	no	yes (10)
		Web-based version	yes	yes	yes	yes	no	yes
**External resources**	Android/iOS application		yes	yes	yes	no	no	no
	Tablet-Smartphone		yes	yes	yes	yes (3)	no	yes (3)
	Smartwatches		yes	yes	yes	no	no	no
	Smartglasses		yes	yes	yes	no	no	no
	Smartpens		yes	yes	yes	no	no	no
**Security and legality**	Data storage	Local	yes	yes	yes	no	yes	yes
		Cloud based	yes	yes	yes	yes	no	yes
		Hybrid (local/cloud based)	yes	yes	yes	no	no	no
		Private server	no	no	yes	yes	yes	no
	Compatibility with regulatory compliance	FDA 21 CFR part 11	no	no	yes (5)	no	yes	yes
		EudraLex Volume 4 Annex 11	no	no	yes (5)	no	yes (6)	yes
	Others	Ability to encrypte text	yes	yes	yes	no	no	no
		Versioning	no	yes	yes	yes	yes	yes
		Ability to sign documents	no	no	no	yes	yes	yes
		**Summary:**	26/42	31/42	39/42	23/42	28/42	30/42

(1) When.pdf file is not inserted in attachment mode.

(2) When file was inserted as a printout.

(3) Only through web version.

(4) Word documents are transformed to.pdf.

(5) When OneNote files are stored under specific Microsoft SharePoint configurations.

(6) Under specific configurations performed by PerkinElmer.

(7) In chemical structures.

(8) Can upload as file attachment or in PDF section

(9) NET client and web version.

(10) Not fully functional.

#### Flexibility

This parameter evaluates the ability of an ELN to allow the users to create their own workflow design. This takes into account how easy/flexible it is to collect data (e.g., drag-and-dropping, file embedding and drawing), organize experiments and projects (e.g., data visualization and hyperlinking) and to edit images or tables. Only OneNote presents all tested subcategories followed by E-Notebook, Elements and Evernote versions (Table 1).

#### Ease of searching

ELN searching options greatly improve the task of finding specific information among notebooks such as DNA sequences, antibodies or protocol-related information. We evaluated this by searching text in projects, experiments, images (Optical Character Recognition) or different type of files (Table 1). OneNote, BIOVIA Notebook and E-Notebook have been able to find text in most of the tested files compared with Elements and both Evernote versions.

#### Collaboration

Academic and pharmaceutical research is often performed within a strong collaborative environment including multiple team members, facilities and internal or external laboratories from different disciplines (e.g., Chemistry, Proteomics) and preferences (Windows, Mac and Linux). Thus ELN’s collaboration and accessibility features are major advantages compared with PLNs and should be prioritized in ELN software development.

In this concern, all ELNs present similar options to easily share information (Table 1). However, concerning accessibility, OneNote, BIOVIA Notebook and Evernote versions could be used in all tested platforms. Although E-Notebook only runs on Windows, virtualization technologies (e.g., VMware or Citrix) are available to allow non-Windows users access software like E-Notebook.

In modern technological environments, software only designed for Windows is less attractive if the use case is to have the broadest possible flexibility on technologies accessing the ELN software system. Elements, being a fully web-based solution, is suitable to be used in all platforms.

#### External resources

This category evaluates the potential of an ELN to be used with external tools such as tablets/smartphones, wearable technology (e.g., smartwatches or smartglasses) or smartpens. These tools could improve the use of an ELN in several manners. Tablets and smartphones connected to smartpens could give the users a physical support to record and visualize data in PLN-like manner. Wearable technology could provide users with immediate data access during experimentation in biosafety level 3 and 4 laboratories or radioactive rooms. Only OneNote and Evernote versions are able to use the aforementioned external tools. Elements, being a fully web-based solution, can be operated in tablets/smartphones but it cannot be connected yet to smartpens or wearable technology (Table 1).

#### Security and legality

ELNs overcome security and legality issues due to their ability to date and archive legible and unchangeable content in a secure and retrievable way. Similarly, ELNs provide options to authenticate experiments with a legally accepted electronic signature. In contrast, PLNs could be easily manipulated to change dates, results or any other content. Here we evaluated the ability of the ELNs to store data in different manners and to provide options to achieve regulatory compliance and other related aspects (Table 1).

Evernote versions, OneNote and BIOVIA Notebook are able to store data locally and in a cloud-based manner. E-Notebook stores data locally while Elements uses cloud storage. However, both programs along with OneNote can store data using private storage servers (Table 1).

A major data security concern lies on the usage of cloud-based systems [11–13]. Data security and privacy protection issues could arise in all stages of data life cycle (Generation → Transfer → Use → Share → Storage → Archival → Destruction) and a comprehensive and integrated security solution is needed [11–13]. In addition, physical access to servers hosting the data is restricted when information is stored *ex situ*. As a result, sensitive data (e.g, Patient-derived information) is at risk from insider attacks. Indeed, The Cloud Security Alliance Report (2016) ranked insider attacks in the top 12 biggest threats to cloud computing [13]. To overcome those problems an on-premise private storage system could be implemented. In this concern, E-Notebook, Elements and OneNote provide this option.

Concerning regulatory compliance, we have evaluated the compatibility of the ELNs to achieve compliance for two international regulations: 1) The Food and Drug Administration's Code of Federal Regulations Title 21 Part 11 (FDA 21 CFR Part 11) and 2) The EudraLex, The Rules Governing Medicinal Products in the European Union, Volume 4, Good Manufacturing Practice Medicinal Products for Human and Veterinary Use, Annex 11: Computerised Systems (EudraLex Volume 4, Annex 11).

FDA 21 CFR Part 11 is a U.S. regulation that sets specifications on electronic records and electronic signatures (ERES). EudraLex Volume 4, Annex 11 is the European equivalent of the FDA 21 CFR Part 11; however, Annex 11 is a guideline not a regulation, as would be Part 11. It is important to notice that Part 11 and Annex 11 apply to medical related industries such as pharmaceutical and biotechnological companies. These regulations do not apply to most academic research institutes.

Evernote versions and Elements do not provide options to reach compliance for any of these regulations. Conversely, E-Notebook has been developed to allow it to be installed, deployed and used in a FDA 21 CFR Part 11 compliant manner. Under specific configurations performed by PerkinElmer, E-Notebook could also reach Annex 11 compliance. BIOVIA Notebook is also compliant with Part 11 and Annex 11.

Downloadable OneNote version is not compliance *per se*; however, OneNote, under specific Microsoft SharePoint 2013 configurations, is able to provide customizable options to achieve compliance. In this regard, Microsoft provides a SharePoint 2013 configuration guidance for Part 11 compliance [14]. However, this document assists towards qualification while compliance is completely up to the implementing party. In addition, software development companies provide services to reach Part 11 and Annex 11 compliance (e.g. Montrium and Paragon Solutions).

With respect to law issues, PLN is considered as the gold standard to protect researchers from legal matters; e.g. intellectual property or fraud accusation. However, an ELN compliant with Part 11 is considered as a legally accepted PLN replacement, even for the stronger regulated pharmaceutical industry. Thus, downloadable OneNote version *per se* is not accepted as a PLN replacement.

Regarding ERES, BIOVIA Notebook, Elements and E-Notebook provide options to electronically sign documents. However, experiments from Evernote and OneNote can be exported *en masse* to.pdf format and signed electronically.

### ELN selection for the survey-based studies

The 42 parameters evaluated in Table 1 do not represent a comprehensive score-based approach to choose the best possible system. Instead, this technical comparison serves as a pointer towards ELN adoption criteria based on the institutional needs. Thus, to further choose two ELNs to be tested in two survey-based studies, we prioritized three subcategories related to specific needs of our institute.

As an academic biomedical research institute, working with patient-derived samples and projects involving sensitive data, we first prioritized “Security/Data storage/Private server” subcategory. In this concern, Evernote versions and BIOVIA Notebook were discarded due to their inability to store data in an on-premise private server. We believe that ELN deployment under an on-premise private storage system will avoid data security issues [11–13] related to public cloud services such as the one provided by Evernote.

Since our institute is structured within a highly collaborative environment including multiple computational platforms (Windows, Mac, Linux), we second prioritized “Collaboration/Accessibility” subcategory. As this study aims to address the usage of tablet-based devices as ELNs complements, we finally prioritized “External resources/Tablet-smartphone” parameter. Thus, we discarded E-Notebook due to its inability to run in non-Windows platforms and tablet-based devices. In addition, most of our computational platforms did not meet the system requirements to run this program and/or its virtualization technologies. We finally selected OneNote and Elements for a proof-of-concept pilot study.

### Survey-based studies

**Fig 1:**
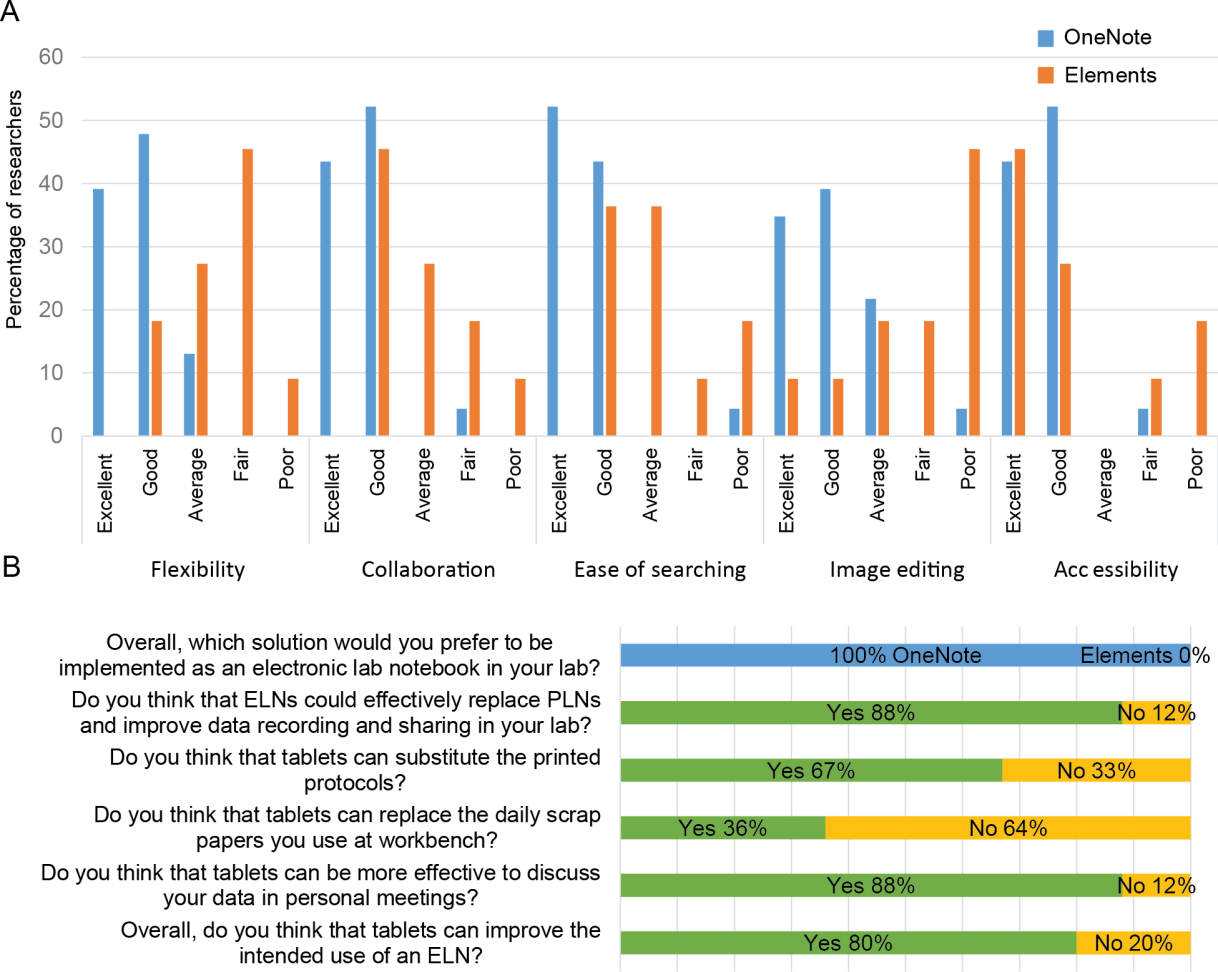
Results of the survey-based study amongst 28 scientists. **(A) Individual questions.** OneNote and Elements features were evaluated by 28 researchers using a 5-point scale (Poor to Excellent). **(B) Group questions.** Histogram showing responses of surveyed researchers concerning ELN selection and implementation and tablet-based devices as ELNs complements.

Two survey-based studies were performed to test Elements and OneNote (See Materials and Methods). First, a group of 28 scientists tested both programs using tablet-based devices as ELNs complements for a 3-month period. Five main parameters (Flexibility, Collaboration, Accessibility, Ease of searching and Image editing) were rated by the volunteers using a 5-point scale (Fig 1A).

Overall, the majority of surveyed scientists rated OneNote as good/excellent in all parameters. Regarding Elements, the majority of volunteers rated Accessibility as good/excellent (72%), Collaboration and Ease of searching parameters as average/good (72%), Flexibility as fair/average (72%) and Image editing as fair/poor (63%) (Fig 1A).

We observed a correlation between the technical evaluation (Table 1) and the responses of surveyed researchers (Fig 1A). For example, OneNote, which presents all Flexibility subcategories (Table 1), was highly evaluated by the respondents (Fig 1A), while Elements, which presents 6 out of 10, was rated as fair/average by 72% of volunteers. Additionally, Elements and OneNote, suitable for all operating system (Table 1), obtained a similar evaluation in Accessibility criteria.

Regarding group questions, 88% of volunteers agreed that ELNs could effectively replace PLNs and improve data recording and sharing (Fig 1B). After discussion with their team members, all surveyed researchers finally preferred OneNote as an ELN solution to be implemented in our biomedical research institute (Fig 1B). This is puzzling considering that Elements was designed as a scientific ELN and OneNote as a general note-taking program. Scientific ELNs have clear advantages over general note-taking programs in standardized experimental settings where experiments are performed routinely with high details on processed data. To understand why researchers preferred OneNote over Elements, we analyzed the data generated during the testing period. We found that all researchers performed non-standardized assays with little experimental details. This could explain why researchers preferred a more flexible solution such as OneNote. However, more research is needed to shed light on this particular aspect.

With respect to the usage of tablet-based devices as ELNs complements, 80% of surveyed researchers reported that those devices improved the usage of ELNs in different manners. For instance, 67% reported that ELN-tablets can substitute printed protocols and 88% reported that those devices are more effective to discuss data in personal meetings compared to PLNs. However, tablets are not appropriate to replace the daily scrap papers used at workbench (Fig 1B).

**Fig 2:**
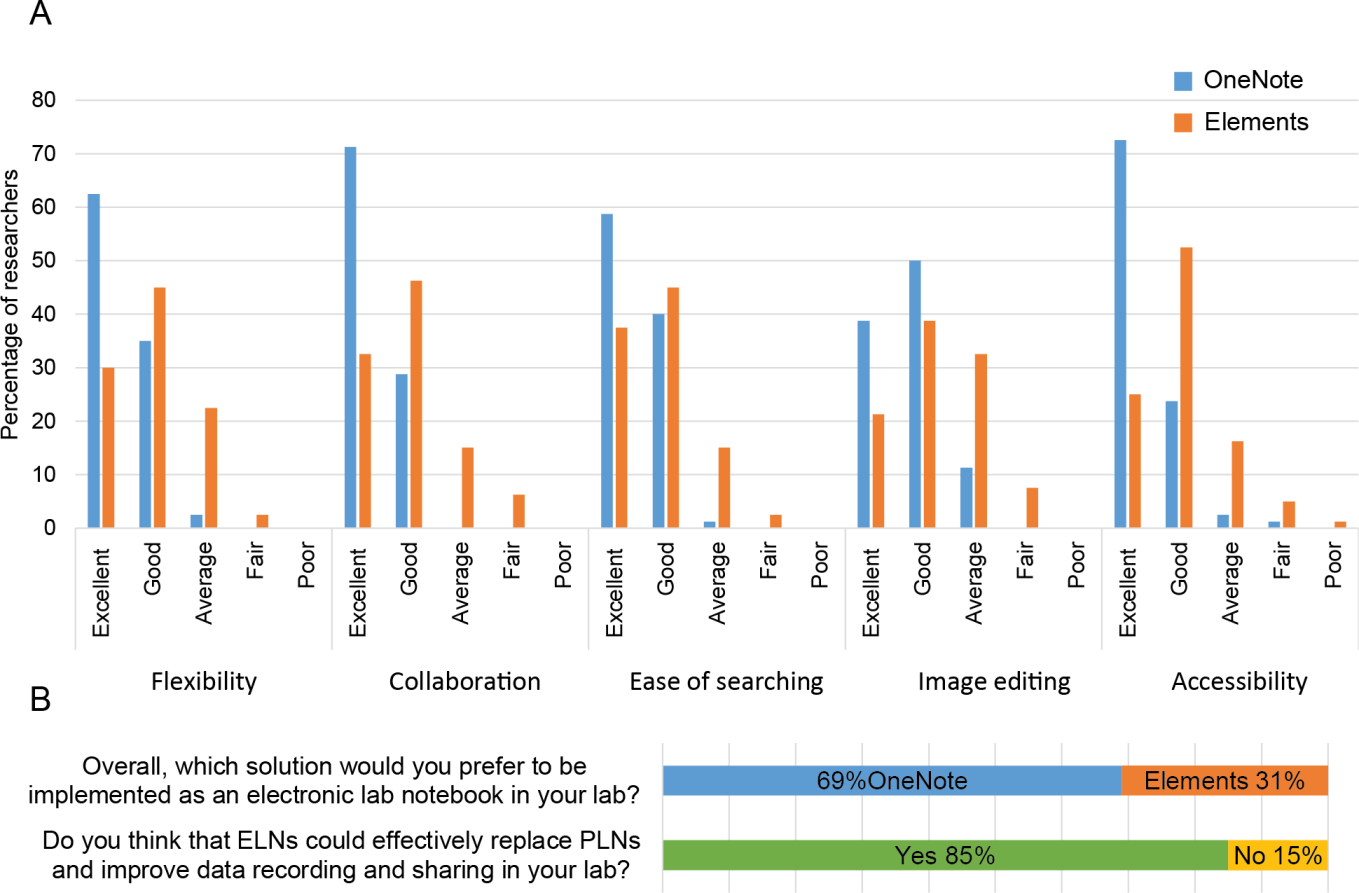
Results of the survey-based study amongst 80 students. **(A)** OneNote and Elements features were evaluated by 80 students using a 5-point scale (Poor to Excellent). **(B)** Histogram showing responses of surveyed students concerning ELN selection and implementation.

Second, an independent group of 80 students evaluated the aforementioned parameters via hands-on practical exercises (See Materials and Methods). Overall, the majority of students rated OneNote as excellent in all parameters except for Image editing, which was evaluated as good (Fig 2A). Regarding Elements, all parameters were rated as good by the majority of the participants. Similar to the first group, the majority of students (69%) preferred OneNote as an ELN solution and 85% agreed that ELNs could effectively replace PLNs and improve data recording and sharing in their laboratories (Fig 2B).

### Wearable technology as ELNs tools

**Fig 3:**
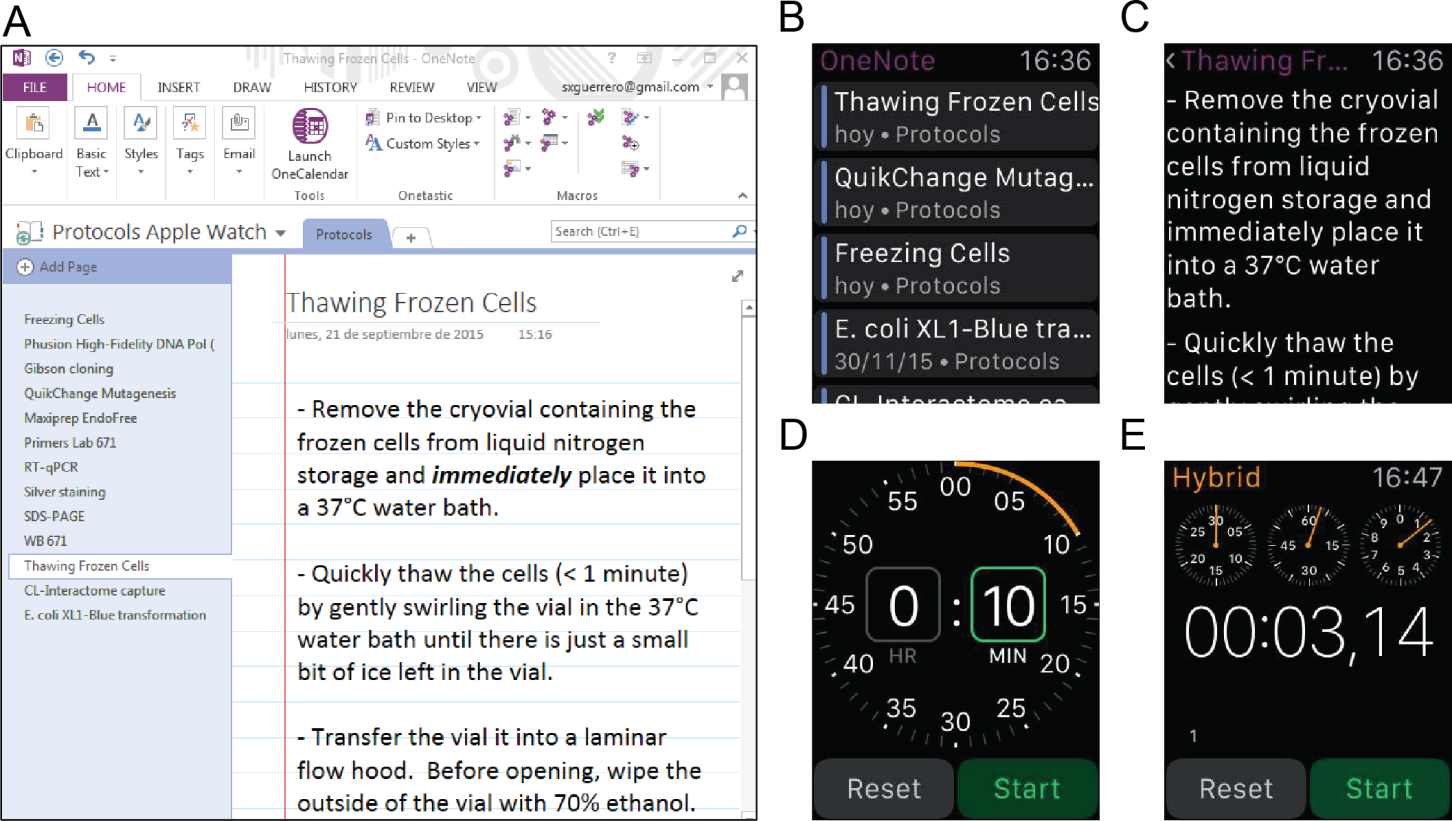
Apple Watch OneNote application as a wearable ELN tool. **(A)** Snapshot of OneNote (Windows version) showing a scientific protocol inside a notebook called “Protocols Apple Watch”. **(B)** Snapshot of Apple Watch OneNote application showing protocols which are synchronized with OneNote Windows version. **(C)** Snapshot of a specific protocol named “Thawing Frozen Cells”. **(D)** Apple Watch timer. **(E)** Apple Watch chronometer.

We finally explored the usage of Apple Watch as a wearable ELN tool using Microsoft OneNote application. This application allows users to have direct access to their notes and visualize data. For instance, users can consult their Protocols during experimentation (Fig 3A–3C); nevertheless, only text-based notes can be visualized. This is a significant advantage when working with large protocols or in places where PLNs are not usually used such as culture or radioactive rooms. Apple Watch can also be used as a substitute for commonly used bench equipment such as timers (Fig 3D) and chronometers (Fig 3E) and provide users with other advantages such as calculators, alarms and reminders.

### Conclusions and Future Perspectives

This work defines important aspects of ELNs characteristics summarized in 5 main parameters that could be used to evaluate other ELNs and to improve their implementation and software development. It is important to notice that these parameters should be prioritized depending of the institutional needs. For example, pharmaceutical laboratories should prioritize regulatory compliance (Part 11 or Annex 11), while most academic institutions do not require these regulations.

ELN security and legality parameters are still a matter of discussion and debate among researchers and should be prioritized in future ELN software development. For instance, options to store data in on-premise private servers would be highly desired to avoid cloud-based security issues [11–13]. Additionally, private clouds services, dedicated to a single organization, are available in the market. These systems along with strong data encryption and stringent security measures could also lead to a secure ELN usage.

Concerning the results of the survey-based studies, researchers and students positively rated Elements and OneNote in all parameters. However, both surveyed groups preferred OneNote as an ELN solution compared to Elements, which was specifically designed for scientific purposes. This tendency towards OneNote could be due to its flexibility of gathering data from non-standardized experiments. Nevertheless, more research is needed to understand this specific aspect. We finally conclude that tablet-based devices and wearable technology such as smart watches could effectively be used as ELN complements.

Over the past 4 years, several startups have emerged as promising innovative software companies offering powerful ELNs (e.g. Benchling, Hivebench and Labfolder, among others). Apart from fulfilling key ELN features, some of them also offer bioinformatics-related tools. For instance, Benchling Lab Notebook presents integrated DNA tools to edit plasmids, design primers and align sequences. In addition, Hivebench, which was recently acquired by Elsevier, can now link Elsevier tools (Pure) and Mendeley Data repositories. Although the potential of these companies is high, their development faces numerous startup-related concerns [15–17]. Despite these challenges, the future appears to be bright for these ELNs.
